# Invasive non-typhoidal Salmonella in sickle cell disease in Africa: is increased gut permeability the missing link?

**DOI:** 10.1186/s12967-018-1622-4

**Published:** 2018-08-30

**Authors:** Seah H. Lim, Barbara A. Methé, Bettina M. Knoll, Alison Morris, Stephen K. Obaro

**Affiliations:** 1 Division of Hematology and Oncology, New York Medical College, Hawthorne, NY USA; 2 Center for Microbiome in Medicine, University of Pittsburgh School of Medicine, Pittsburgh, USA; 3 Division of Infectious Diseases, New York Medical College, Hawthorne, NY USA; 4 Department of Pediatrics, University of Nebraska Medical Center, Omaha, NE USA; 5 Westchester Medical Center Cancer Institute, 19 Bradhurst Avenue, Suite 2575S, Hawthorne, NY 10532 USA

**Keywords:** Sickle cell disease, Invasive non-typhoidal Salmonella, Intestinal dysbiosis, Gut permeability

## Abstract

Non-typhoidal Salmonella usually induces self-limiting gastroenteritis. However, in many parts of Africa, especially in individuals who are malnourished, infected with malaria, or have sickle cell disease, the organism causes serious and potentially fatal systemic infections. Since the portal of entry of non-typhoidal Salmonella into the systemic circulation is by way of the intestine, we argue that an increased gut permeability plays a vital role in the initiation of invasive non-typhoidal Salmonella in these patients. Here, we will appraise the evidence supporting a breach in the intestinal barrier and propose the mechanisms for the increased risks for invasive non-typhoidal Salmonella infections in these individuals.

## Background

Sickle cell disease (SCD) is a major global hemoglobinopathy and affects between 20 and 25 million people worldwide, with an incidence of approximately 300,000 births/year [1]. It is particularly prevalent in the African continent, with nearly 80% of the SCD births occurring in sub-Saharan Africa [2]. It is a chronic illness and affected individuals suffer from recurrent vaso-occlusive crises (VOC) crises, poor quality of life, and a shortened lifespan. If life-span extends into adulthood, end-organ damage occurs in these patients, affecting the kidneys, brains, lungs, and eyes. The life expectancy of SCD in the United States (US) has increased to 42 and 48 years for men and women, respectively [3]. However, 50–80% of children with SCD in Africa still die before the age of 5 years [4]. Since many babies are born and die outside of hospital, it is likely that the mortality rate due to SCD in African children is much higher [5, 6].

While infections caused by encapsulated bacterial agents are the most widely recognized cause of life threatening infections in SCD, specific species vary across geographic regions. In Europe and the US, *Streptococcus pneumoniae* is the leading cause but in Africa, enteric bacteria, such as *Salmonella* are most common. Pneumococcal infections can be readily prevented with penicillin prophylaxis and the advent of pneumococcal conjugate vaccines has been a major breakthrough in disease prevention. Although a typhoid fever/invasive non-Typhoidal *Salmonella* (iNTS) disease conjugate vaccine targeting *S.* enteritidis, *S.* typhimurium, and *S.* typhi is currently in Phase 1 clinical trials, prevention of Salmonella infections, particularly those by NTS, remains a major challenge. Thus, improved understanding of the pathogenesis of iNTS warrants urgency to provide new tools for preventive care of SCD in populations most afflicted by the infections.

In this paper, we will examine the evolving data supporting a breach of gut permeability in SCD. A compromised gut barrier may facilitate the portal of entry for iNTS in these patients. We will propose potential preventive strategies to reduce the risk for iNTS in this group of patients.

## Main text

### Public health impact of non-typhoidal *Salmonella*

NTS is among the three most common pathogens causing systemic infections in children and adults in the sub-Saharan Africa [7, 8]. NTS consists of many serovars, with *S.* typhimurium being the serovar that is the most commonly implicated pathogen. Unlike typhoidal *Salmonella* that consists of the serovars Typhi and Paratyphi and causes the systemic disease of typhoid, NTS generally induces self-limited gastroenteritis in human. However, in many parts of Africa, NTS causes highly significant invasive systemic infections [9, 10]. The clinical features of invasive NTS (iNTS) are distinct from those of gastroenteritis or typhoid disease. These patients usually present with nonspecific fever similar to malaria, and in some patients, pneumonia, meningitis or osteomyelitis. The impact of iNTS on childhood mortality exceeds malaria in some African communities [11]. The estimated mortality rates for iNTS among hospitalized patients in Africa ranges from 4.4 to 27% for children [12–14] and 22 to 47% for adults [15, 16]. The mortality rate is highest in those with meningitis and is higher than any other common bacterial causes of meningitis. In Malawi, the mortality rate due to NTS meningitis in the neonates was 64%, compared to 26% in those with Group B Streptococcal meningitis [17]. The burden due to iNTS is significant. For example, it has been estimated that iNTS occurred in 88 cases per 100,000 person-years in the age group of 5 years old in rural Kenya, while in Mozambique, NTS accounted for 120 cases per 100,000 person-years [17]. These incidences are likely grossly under-estimated since many children with iNTS died before reaching the local hospitals [8, 11].

The use of whole genome sequencing has become important for monitoring the prevalence, movement and genotype of infectious disease agents such as *Salmonella*. Sequence analysis of invasive *S*. typhimurium from Malawi and Kenya identified a dominant type, designated ST313, which is rarely isolated outside of Africa [18]. Whole-genome sequencing of ST313 NTS found genetic element encoding multi-drug resistance (MDR) genes located on a virulence-associated plasmid of the organism. Unfortunately, the factors contributing to the high prevalence of iNTS remain poorly defined. Our surveillance platform of 9345 children in Kano, Nigeria, identified that the age-adjusted odds ratio for clinically significant iNTS was much higher in SCD than those without the disease (OR 4.28, 95% CI 2.3–7.9) [19, 20]. We have also previously shown that SCD patients have alteration of their lymphocyte phenotype and functions [21]. In addition to splenic dysfunction associated with SCD, children with malnutrition, malaria, and human immunodeficiency virus (HIV) are also more susceptible to iNTS [10, 22]. However, these immunocompromised states only explain the obstacles in eradicating micro-organisms that successfully enter the blood stream and do not address the disproportionally higher incidence of enteric-derived systemic infections in these patients, unless there is a breach in the gut permeability in these patients.

### Regulation of gut permeability

**Fig. 1:**
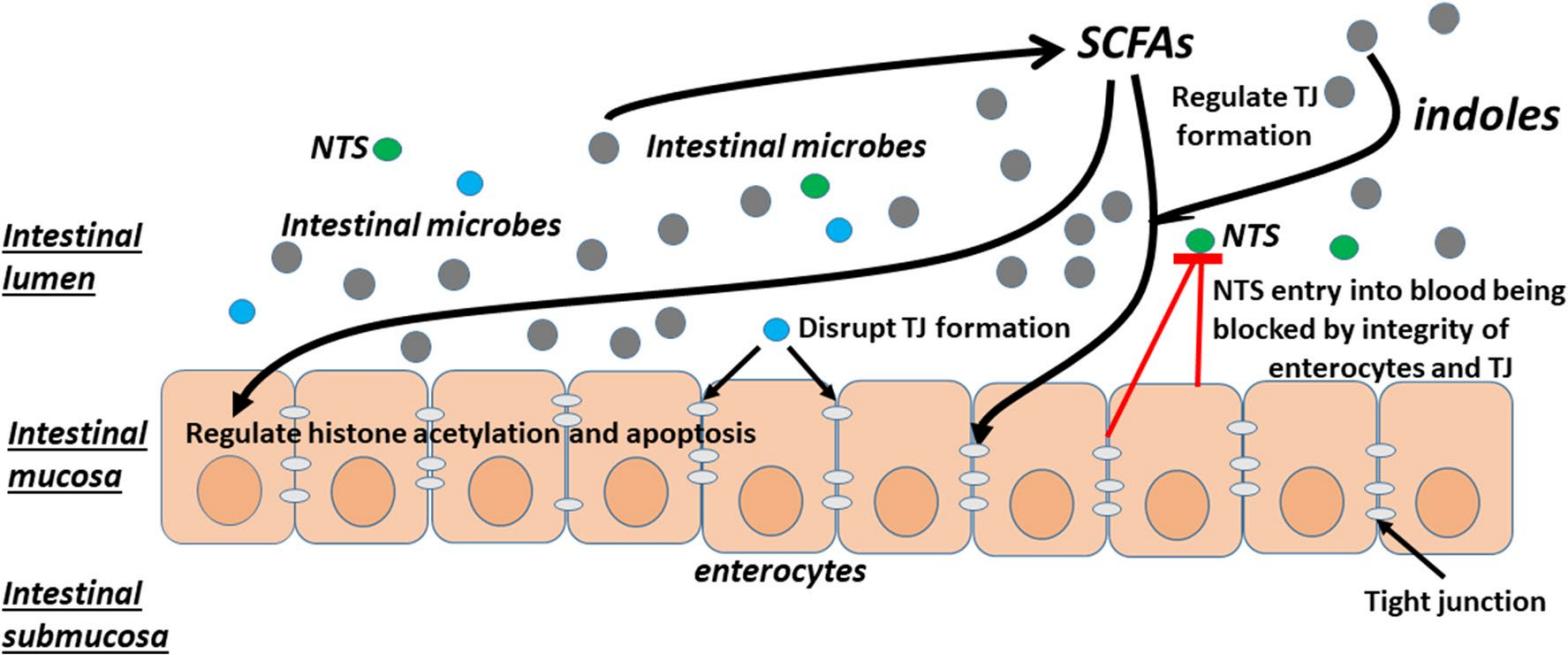
Intestinal homeostasis provided by a balanced intestinal microbiota community. A balanced intestinal microbiota community helps maintain microbial homeostasis and immunologic tolerance, and modulate the metabolic processes that influence the intestinal permeability. An intact intestinal barrier is provided by an anatomical barrier of the intestinal wall and a physiological barrier linked to the intestinal microbiota and elements of the mucosal immune system. The space between enterocytes is sealed by TJs that regulate the flow of water ions and small molecules across the barrier. TJ development is maintained by SCFAs and indole metabolites produced by some intestinal microbes. However, TJ development can also be disrupted by the relative abundance of certain intestinal microbes. An intact intestinal barrier prevents the translocation of intestinal microbes, including NTS, across the barrier into the systemic circulation, thereby reduce the risks for systemic infections by the enteric microbes

Gut permeability is a complex system provided by an anatomical barrier of the intestinal wall and a physiological barrier closely linked to the intestinal microbiota and elements of the mucosal immune system [23]. The intercellular space between enterocytes is sealed by tight junctions (TJs) that regulate the flow of water ions and small molecules. TJs are composed of proteins such as claudins, occludin, and tricellin. A balanced intestinal microbiota community not only helps maintain the microbial homeostasis and immunologic tolerance, but also modulates the metabolic processes that influence the intestinal permeability. This can occur due to effects on the production of short chain fatty acids (SCFAs) that play an important role in enterocyte development [24, 25] or through bacterial factors that directly affect the development of TJs between enterocytes [26–34] (Fig. 1). Butyrate, a SCFA, promotes intestinal barrier function, increases trans-epithelial electrical resistance and decreases inulin permeability [35, 36]. Reduced levels of butyrate occurred in mucosal tissue are associated with decreased histone acetylation and increased enterocyte apoptosis [36]. Indole metabolites produced from tryptophan by some enteric microbes also provide protection against enterocyte injury by modulating the host-microbe homeostasis at the mucosal surface. Indole metabolites have also been found in mice to modulate incretin secretion from colonic L cells [37] and increases epithelial tight-junction resistance [38]. It is, therefore, not surprising that intestinal dysbiosis may result in increased gut permeability and decreased enterocyte health and is implicated in the pathogenesis of extra-colonic diseases.

### Factors causing intestinal dysbiosis in Africa

#### Diarrheal illnesses affecting intestinal microbiota compositions

Diarrheal illnesses are common in Africa and may impact the gut microbiome composition and lead to mucosal damage. Most of the diarrhea-related deaths in children are due to unsafe water, inadequate sanitation, and insufficient hygiene [39, 40]. Increased motility associated with diarrhea per se has also been found to alter the intestinal microbiome, characterized by striking difference in the stool and mucosal microbiotas, with *Firmicutes* being found predominantly on the mucosa and *Bacteroidetes* in the stools [41]. It also results in relative shifts in the phyla *Bacteroidetes* and *Firmicutes* and to a relative increase in *Proteobacteria* on the mucosa, a finding commonly seen in inflammatory bowel disease [41]. Frequent diarrheal illnesses that induce rapid colonic transit, worsened in some cases by mucosal inflammation induced by the infectious agents, would not only cause mucosal damage but also changes in the intestinal metabolomics involved in normal enterocyte health and TJ formation.

#### Malnutrition affecting intestinal microbiota compositions

The African continent has a high prevalence of malnutrition [42], and malnutrition has been linked to alteration in the gut microbiome. It is a major problem and sets up a vicious cycle of impaired immunity, increased risks for infections, and worsening malnutrition, especially in children with SCD who already have chronic ill health due to SCD. Malnutrition affects the intestinal microbiota compositions [43] and may further affect food intake metabolism. Balanced nutrition is needed for enterocyte health [44] and impaired enterocyte development affects intestinal permeability [43]. Malnutrition, therefore, not only affects immunity against infection, but also allows enhanced translocation of enteric bacteria into the systemic circulation due to a breach of the intestinal barrier.

#### Malaria

NTS bacteremia overlaps significantly with malaria in Africa, both in terms of seasonality and affected age groups. Several studies have demonstrated parallel decreases in incidence of malaria and NTS bacteremia in the same geographical area over time [45]. For example, a comparative study of the temporal trends of childhood malaria and NTS infection from two locations in the Gambia at three-time points between 1979 and 2005 evaluated the percentage of malaria positive outpatient thick blood films and the percentage of admissions associated with malaria over time. The estimated incidence of NTS infection at the coastal site fell from 60 (1979–1984) to 10 (2003–2005) cases per 100,000-person years and the proportion of outpatients with suspected malaria who were parasitemic fell in parallel from 33% in 1999 to 6% in 2007, and malaria-associated hospital admissions from 14.5% in 1999 to 5% in 2007. At the second location, in the hinterland, the estimated incidence of NTS infection fell from 105 per 100,000-person years between 1989 and 1991, to 29 in 2008 cases mirrored the drop in the prevalence of malaria parasitemia from 45% in 1992 to 10% in 2008. These drops in the incidence cannot be explained purely by any change in healthcare since the incidence of pneumococcal bacteremia at both sites remained the same during these periods [46]. Many mechanisms have been proposed to explain how malaria causes susceptibility to NTS, although the most consistent evidence is that malarial hemolysis creates conditions which favor bacterial growth, by increasing iron availability and by impairing neutrophil function [47], thereby preventing the effective eradication of NTS that successfully enter the systemic blood stream via the intestine. Whether or not malaria infections facilitate the entry of NTS into the blood stream remains speculative. There are two possible mechanisms whereby malaria infections enhance NTS translocation across the intestinal barrier. First, chronic malaria and parasitemia induces a state of anorexia and malnutrition that might affect healthy enterocyte development [43] and balanced intestinal microbiota composition [43] needed to maintain gut permeability. Second, previous studies have found that malaria-infected erythrocytes are sequestrated in various capillary beds [48] and induce local hypoxemia. In SCD patients, local tissue hypoxemia is made worse by erythrocyte sickling induced by the sequestrated erythrocytes. The resultant hypoxemia will affect not only normal enterocyte development, but also induce intestinal dysbiosis [49] that may impair TJ formation and the production of SCFAs needed for enterocyte health.

#### Human immunodeficiency virus infection

HIV is prevalent in Africa. Intestinal dysbiosis occurs frequently in HIV patients, especially before the initiation of anti-retroviral therapy [50]. The consistent findings in these patients include the depletion of Bacteroides and enrichment of Proteobacteria [51–53]. Bacteroides are associated with modulating intestinal inflammation and Proteobacteria with pro-inflammatory responses. Intestinal dysbiosis has been associated with increased microbial translocation and monocyte activation markers, and inferior disease outcome [54]. The increased microbial translocation suggests a breach in the intestinal permeability.

### The effects of SCD on intestinal microbiota compositions

SCD per se is associated with intestinal dysbiosis. We have documented that pediatric and adult patients with SCD in the US showed altered intestinal microbiota compositions, with significantly lower abundance of *Pseudobutyrivibrio* and *Alistipes* in SCD patients compared to subjects with sickle trait [55]. These organisms negatively correlated with serum lactate dehydrogenase, a marker of hemolysis. We also found that *Lachnoclostridium* positively correlated with higher baseline hemoglobin and fetal hemoglobin and lower baseline C-reactive protein in SCD patients. The underlying cause for the dysbiosis is currently unclear, but is most likely due, at least in part, to the hypoxemia induced by recurrent sickling in the splanchnic vasculature. Hypoxia alters intestinal microbiota communities [49]. There is indirect evidence supporting the occurrence of vaso-occlusive crisis in the splanchnic vessels and causing intestinal hypoxemia, e.g. the occurrence of ischemic colitis in SCD [56, 57]. The propensity for the splenic artery, part of the splanchnic vasculature, of children with SCD to develop atherosclerosis [58] further supports the notion that VOC occurs in the intestinal vasculature. The dysbiosis resulting from hypoxemia may, therefore, result in a breach in the gut permeability.

### What is the evidence supporting increased gut permeability in SCD?

Previous clinical and laboratory studies have raised the concept of increased gut permeability in SCD. SCD patients have higher baseline total white cell counts than those with hemoglobin (Hb) AA phenotype [59]. Their neutrophils are also more likely to be activated, as shown by the higher expression of activation molecules, e.g. CD64 [60] and CD11b/CD18 [61], and elevated levels of soluble CD62L, a serum marker of in vivo neutrophil activation [60]. Neutrophils are pivotal in the initiation and propagation of VOC. In SCD mice, sickled erythrocytes more commonly adhered to activated neutrophils than to endothelium [62]. These immobilized neutrophils act as niduses for sickled erythrocytes to attach to and cause VOC. A study found that the quality and quantity of circulating aged neutrophils are regulated by Toll-like receptor (TLR) 2, TLR 4, and Myd88 [63]. Mice genetically engineered to not express TLR 2, TLR 4, or Myd88 had lower numbers of circulating activated neutrophil. Furthermore, SCD mice treated with a combination of ampicillin, neomycin, vancomycin, and metronidazole had a decrease in the number of activated neutrophils and were protected from fatal tumor necrosis factor (TNF) α-induced VOC [63]. The most common cause for an increase in the number and activation of neutrophils is an innate immune response from the release of inflammatory cytokines following receptor recognition of pathogen-associated molecular patterns (PAMPs). TLR and Myd88 are well-recognized receptors for PAMPs [64, 65]. A compromised gut permeability that allows increased translocation of intestinal bacteria into the bloodstream where the microbes or their products encounter neutrophils [66] could explain why SCD patients have higher baseline levels of circulating aged neutrophils and could also explain the higher incidence and severity of iNTS among SCD patients compared to those without the disease in the African continent.

### Proposed mechanisms for increased iNTS in African SCD

**Fig. 2:**
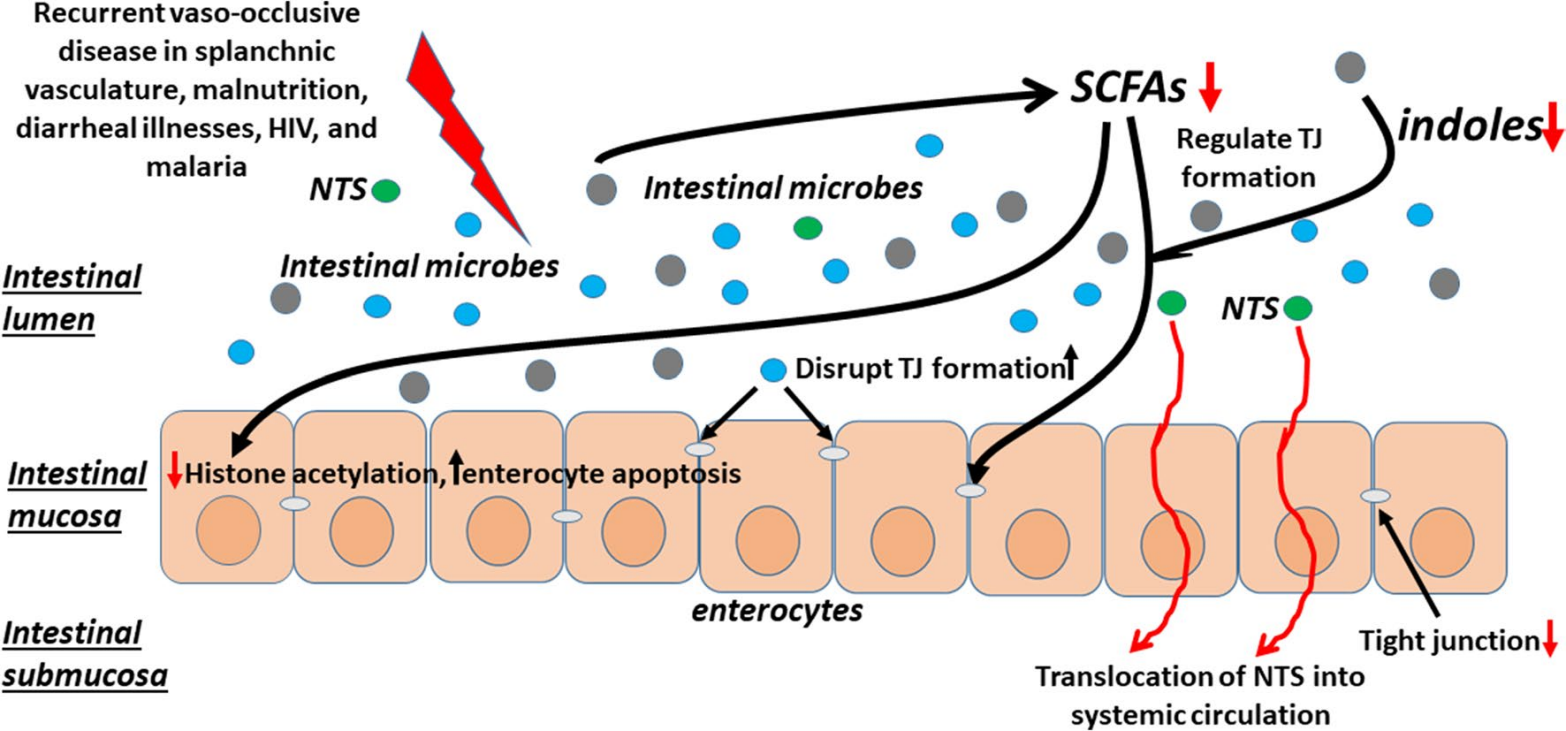
Intestinal dysbiosis leads to a breakdown of the normal gut barrier. Due most likely to the intermittent hypoxia induced by recurrent vaso-occlusive crises of the splanchnic vasculature, patients with SCD often experience intestinal dysbiosis. However, frequent diarrheal illnesses, malnutrition, and malaria further worsen the intestinal dysbiosis that may result in changes in the compositions of the intestinal microbes disrupt TJ formation and reductions in the production of SCFAs that enhance TJ formation and enterocyte health, while reduce enterocyte apoptosis. Deficiencies of the indole metabolites produced by microbial metabolism of tryptophan, enterocyte health is further compromised. A breach in the intestinal barrier results in an increased in gut permeability, enhancing translocation of enteric NTS and other microbes to cause systemic infections

Based on the above considerations, we propose the following model for the initiation and entry of iNTS into the systemic circulation in SCD (Fig. 2). In the setting of an intact gut barrier, patients exposed to NTS are protected from iNTS by an intact mucosa formed by healthy enterocytes maintained by indole metabolites, and by the presence of effective TJs between enterocytes promoted by normal intestinal microbiota and SCFAs. However, a combination of frequent diarrheal illnesses, malnutrition, HIV, and malaria in some of these patients render a change in the intestinal microbiota. These factors are further worsened in patients with SCD whose gut barrier has already been compromised due to the disease. As a result, the microbes capable of disrupting TJ formation are increased, causing a deficiency of TJs between enterocytes and an imbalance of the indole metabolites produced by the microbes. Changes in the composition of the intestinal microbiota also result in changes in the metabolomics and cause a reduction in the production of SCFAs. The consequences of a deficiency of SCFAs include reduced histone acetylation in the enterocytes, increased enterocyte apoptosis, and dysregulation of TJ formation. The combination of a subclinical damaged intestinal mucosa, due to increased enterocyte apoptosis and reduced indole metabolites, and an increased permeability provides an optimal entry point for intestinal NTS to cause systemic diseases in these SCD patients.

## Conclusions

### Looking into the future

Patients with sickle cell disease in Africa are at higher risk for developing invasive non-typhoidal Salmonella infections, such as meningitis and osteomyelitis, than those without sickle cell disease in the same geographical locations or with sickle cell disease in developed countries. However, specific interventions to reduce the burden of disease continues to be hampered by poor understanding of the pathogenesis of the infections caused by these bacteria, which for the most part are commensals of the gut and only cause self-limiting gastrointestinal symptoms in developed countries. Understanding the epidemiology of the gut microbiome in the tropics will provide insights into new approaches for reducing the incidence of invasive enteric bacterial infections. A breach in the intestinal permeability may play an important role in the pathogenesis of invasive NTS infections in these patients since the portal of entry of the microbes into the systemic circulating is the intestine. A breakdown in the gut barrier in these patients may occur due to intestinal dysbiosis induced by recurrent sickle cell vaso-occlusive crises in the splanchnic vasculature, frequent diarrheal illnesses, malaria, and malnutrition. Based on the mechanisms we have proposed here, since intestinal-protective effect may be conferred by the indole metabolites produced by intestinal commensal bacteria, it would be appropriate to investigate the role of microbiota-based therapeutic approaches in African SCD children to prevent iNTS. Restoration or preservation of intestinal commensal bacteria by probiotics or prebiotics, especially in African SCD children, may provide the bridge to reduce the incidence of iNTS.

## Abbrevations

Hb: hemoglobin
HIV: human immunodeficiency virus
iNTS: invasive nontyphoidal Salmonella
NTS: nontyphoidal Salmonella
PAMP: pathogen-associated molecular patterns
SCD: sickle cell disease
SCFA: short chain fatty acid
TJ: tight junction
TLR: toll-like receptor
TNF: tumor necrosis factor
US: United States
VOC: vaso-occlusive crisis

## References

[1] Piel FB, Patil AP, Howes RE (2013). Global epidemiology of sickle haemoglobin in neonates: a contemporary geostatistical model-based map and population estimates. Lancet.

[2] World Health Organization (1994). Guidelines for the control of haemoglobin disorders.

[3] Makani J, Cox SE, Soka D (2011). Mortality in sickle cell anemia in Africa: a prospective cohort study in Tanzania. PLoS ONE.

[4] Platt OS, Brambilla DJ, Rosse WF (1994). Mortality in sickle cell disease. Life expectancy and risk factors for early death. N Engl J Med.

[5] Aygun B, Odame I (2012). A global perspective on sickle cell disease. Pediatr Blood Cancer.

[6] Serjeant GR (2005). Mortality from sickle cell disease in Africa. BMJ.

[7] Shaw AV, Reddy EA, and Crump JA. Etiology of community-acquired bloodstream infections in Africa [abstract L-620]. In: Program and abstracts of the 46th Annual Meeting of the Infectious Diseases Society of America (Washington, DC). Alexandria: Infectious Diseases Society of America, 2008.

[8] Sigauque B, Roca A, Mandomando I (2009). Community-acquired bacteremia among children admitted to a rural hospital in Mozambique. Pediatr Infect Dis J.

[9] Reddy EA, Shaw AV, Crump JA (2010). Community-acquired bloodstream infections in Africa: a systematic review and meta-analysis. Lancet Infect Dis.

[10] Graham SM (2010). Nontyphoidal salmonellosis in Africa. Curr Opin Infect Dis.

[11] Berkley JA, Lowe BS, Mwangi I (2005). Bacteremia among children admitted to a rural hospital in Kenya. N Engl J Med.

[12] Brent AJ, Oundo JO, Mwangi I, Ochola L, Lowe B, Berkley JA (2006). Salmonella bacteremia in Kenyan children. Pediatr Infect Dis J.

[13] Walsh AL, Phiri AJ, Graham SM, Molyneux EM, Molyneux ME (2000). Bacteremia in febrile Malawian children: clinical and microbiologic features. Pediatr Infect Dis J.

[14] Graham SM, Walsh AL, Molyneux EM, Phiri AJ, Molyneux ME (2000). Clinical presentation of non-typhoidal Salmonella bacteraemia in Malawian children. Trans R Soc Trop Med Hyg.

[15] Gordon MA, Banda HT, Gondwe M (2002). Non-typhoidal salmonella bacteraemia among HIV-infected Malawian adults: high mortality and frequent recrudescence. AIDS.

[16] Gordon MA, Graham SM, Walsh AL (2008). Epidemics of invasive *Salmonella enterica* serovar enteritidis and *S. enterica* serovar typhimurium infection associated with multidrug resistance among adults and children in Malawi. Clin Infect Dis.

[17] Milledge J, Calis JC, Graham SM (2005). Aetiology of neonatal sepsis in Blantyre, Malawi: 1996–2001. Ann Trop Paediatr.

[18] Kingsley RA, Msefula CL, Thomson NR (2009). Epidemic multiple drug resistant Salmonella Typhimurium causing invasive disease in sub-Saharan Africa have a distinct genotype. Genome Res.

[19] Obaro S, Lawson L, Essen U (2011). Community acquired bacteremia in young children from central Nigeria–a pilot study. BMC Infect Dis.

[20] Obaro SK, Hassan-Hanga F, Olateju EK (2015). Salmonella bacteremia among children in central and northwest Nigeria, 2008–2015. Clin Infect Dis.

[21] Balandya E, Reynolds T, Obaro S, Makani J (2016). Alteration of lymphocyte phenotype and function in sickle cell anemia: implications for vaccine responses. Am J Hematol.

[22] Berkley JA, Bejon P, Mwangi T (2009). HIV infection, malnutrition, and invasive bacterial infection among children with severe malaria. Clin Infect Dis.

[23] Bischoff SC, Barbara G, Buurman W (2014). Intestinal permeability—a new target for disease prevention and therapy. BMC Gastroenterol.

[24] Malago JJ, Koninkx JF, Douma PM (2003). Differential modulation of enterocyte-like Caco-2 cells after exposure to short-chain fatty acids. Food Addit Contam.

[25] Goverse G, Molenaar R, Macia L (2017). Diet-derived short chain fatty acids stimulate intestinal epithelial cells to induce mucosal tolerogenic dendritic cells. J Immunol.

[26] Maslowski KM, Vieira AT, Ng A (2009). Regulation of inflammatory responses by gut microbiota and chemoattractant receptor GPR43. Nature.

[27] Chassaing B, Darfeuille-Michaud A (2011). The commensal microbiota and enteropathogens in the pathogenesis of inflammatory bowel diseases. Gastroenterology.

[28] Chow J, Tang H, Mazmanian SK (2011). Pathobionts of the gastrointestinal microbiota and inflammatory disease. Curr Opin Immunol.

[29] Ganesh BP, Klopfleisch R, Loh G, Blaut M (2013). Commensal Akkermansia muciniphila exacerbates gut inflammation in Salmonella Typhimurium-infected gnotobiotic mice. PLoS ONE.

[30] Amieva MR, Vogelmann R, Covacci A, Tompkins LS, Nelson WJ, Falkow S (2003). Disruption of the epithelial apical-junctional complex by *Helicobacter pylori* CagA. Science.

[31] Bagnoli F, Buti L, Tompkins L, Covacci A, Amieva MR (2005). *Helicobacter pylori* CagA induces a transition from polarized to invasive phenotypes in MDCK cells. Proc Natl Acad Sci U S A.

[32] Saadat I, Higashi H, Obuse C (2007). *Helicobacter pylori* CagA targets PAR1/MARK kinase to disrupt epithelial cell polarity. Nature.

[33] Wroblewski LE, Shen L, Ogden S (2009). *Helicobacter pylori* dysregulation of gastric epithelial tight junctions by urease-mediated myosin II activation. Gastroenterol.

[34] Lapointe TK, O’Connor PM, Jones NL, Menard D, Buret AG (2010). Interleukin-1 receptor phosphorylation activates Rho kinase to disrupt human gastric tight junctional claudin-4 during *Helicobacter pylori* infection. Cell Microbiol.

[35] Peng L, He Z, Chen W, Holzman IR, Lin J (2007). Effects of butyrate on intestinal barrier function in a Caco-2 cell monolayer model of intestinal barrier. Pediatr Res.

[36] Matthewson ND, Jenq R, Matthew AV (2016). Gut microbiome-derived metabolites modulate intestinal epithelial cell damage and mitigate graft-versus-host disease. Nat Immunol.

[37] Chimerel C, Emery E, Summers DK, Keyser U, Gribble FM, Reimann F (2014). Bacterial metabolite indole modulates incretin secretion from intestinal enteroendocrine L cells. Cell Rep.

[38] Bansal T, Alaniz RC, Wood TK, Jayaramana A (2010). The bacterial signal indole increases epithelial-cell tight-junction resistance and attenuates indicators of inflammation. Proc Natl Acad Sci.

[39] UNICEF (2006). Progress for children: a report card on water and sanitation.

[40] Black RE, Morris S, Bryce J (2003). Where and why are 10 million children dying every year?. Lancet.

[41] Gorkiewicz G, Thallinger GG, Trajanoski S (2013). Alterations in the colonic microbiota in response to osmotic diarrhea. PLoS ONE.

[42] Ndemwa M, Wanyua S, Kaneko S, Karama M, Anselimo M (2017). Nutritional status and association of demographic characteristics with malnutrition among children less than 24 months in Kwale County, Kenya. Pan Afr Med J.

[43] Kane AV, Dinh DM, Ward HD (2015). Childhood malnutrition and the intestinal microbiome malnutrition and the microbiome. Pediatr Res.

[44] Singh RK, Chang HW, Yan D (2017). Influence of diet on the gut microbiome and implications for human health. J Transl Med.

[45] Takem EN, Roca A, Cunnington A (2014). The association between malaria and nontyphoid Salmonella bacteraemia in children in sub-Saharan Africa: a literature review. Malar J.

[46] Mackenzie G, Ceesay SJ, Hill PC (2010). A decline in the incidence of invasive non-typhoidal Salmonella infection in The Gambia temporally associated with a decline in malaria infection. PLoS ONE.

[47] van Santen S, de Mast Q, Swinkels DW, van der Ven AJAM (2013). The iron link between malaria and invasive non-typhoid Salmonella infections. Trends Parasitol.

[48] Cunnington AJ, Riley EM, Walther M (2013). Stuck in a rut? Reconsidering the role of parasite sequestration in severe malaria syndromes. Trends Parasitol.

[49] Moreno-Indias I, Torres M, Montserrat JM (2015). Intermittent hypoxia alters gut microbiota diversity in a mouse model of sleep apnoea. Eur Respir J.

[50] Tincati C, Douek DC, Marchetti G (2016). Gut barrier structure, mucosal immunity and intestinal microbiota in the pathogenesis and treatment of HIV infection. AIDS Res Ther.

[51] Vujkovic-Cvijin I, Dunham RM, Iwai S (2013). Dysbiosis of the gut microbiota is associated with HIV disease progression and tryptophan catabolism. Sci Transl Med.

[52] Dillon SM, Lee EJ, Kotter CV (2014). An altered intestinal mucosal microbiome in HIV-1 infection is associated with mucosal and systemic immune activation and endotoxemia. Mucosal Immunol.

[53] Mutlu EA, Keshavarzian A, Losurdo J (2014). A compositional look at the human gastrointestinal microbiome and immune activation parameters in HIV infected subjects. PLoS Pathog.

[54] Bandera A, de Benedetto I, Bozzi G, Gori A (2018). Altered gut microbiome composition in HIV infection: causes, effects and potential intervention. Curr Opin HIV AIDS.

[55] Lim SH, Morris A, Li K (2018). Intestinal microbiome analysis revealed dysbiosis in sickle cell disease. Am J Hematol.

[56] Gage TP, Gagnier JM (1983). Ischemic colitis complicating sickle cell crisis. Gastroenterol.

[57] Karim A, Ahmed S, Rossoff LJ, Siddiqui R, Fuchs A, Multz AS (2002). Fulminant ischaemic colitis with atypical clinical features complicating sickle cell disease. Postgrad Med J.

[58] de Chadarevian JP, Balarezo FS, Heggere M, Dampier C (2001). Splenic arteries and veins in pediatric sickle cell disease. Pediatr Dev Pathol.

[59] Anyaegbu CC, Okpala IE, Akren’Ova YA, Salimonu LS (1998). Peripheral blood neutrophil count and candidacidal activity correlate with the clinical severity of sickle cell anaemia (SCA). Eur J Haematol.

[60] Lard LR, Mul FP, de Haas M, Roos D, Duits AJ (1999). Neutrophil activation in sickle cell disease. J Leukoc Biol.

[61] Lum AF, Wun T, Staunton D, Simon SI (2004). Inflammatory potential of neutrophils detected in sickle cell disease. Am J Hematol.

[62] Turhan A, Weiss LA, Mohandas N, Coller BS, Frenette PS (2002). Primary role for adherent leukocytes in sickle cell vascular occlusion: a new paradigm. Proc Natl Acad Sci U S A.

[63] Zhang D, Chen G, Manwani D (2015). Neutrophil ageing is regulated by the microbiome. Nature.

[64] Tartey S, Takeuchi O (2017). Pathogen recognition and toll-like receptor targeted therapeutics in innate immune cells. Int Rev Immunol.

[65] Wiens M, Korzhev M, Krasko A (2005). Innate immune defense of the sponge *Suberites domuncula* against bacteria involves a MyD88-dependent signaling pathway. Induction of a perforin-like molecule. J Biol Chem.

[66] Lim SH, Fast L, Morris A (2016). Sickle cell vaso-occlusive crisis: it’s a gut feeling. J Transl Med.

